# *Porphyromonas gingivalis* Facilitates the Development and Progression of Destructive Arthritis through Its Unique Bacterial Peptidylarginine Deiminase (PAD)

**DOI:** 10.1371/journal.ppat.1003627

**Published:** 2013-09-12

**Authors:** Katarzyna J. Maresz, Annelie Hellvard, Aneta Sroka, Karina Adamowicz, Ewa Bielecka, Joanna Koziel, Katarzyna Gawron, Danuta Mizgalska, Katarzyna A. Marcinska, Malgorzata Benedyk, Krzysztof Pyrc, Anne-Marie Quirke, Roland Jonsson, Saba Alzabin, Patrick J. Venables, Ky-Anh Nguyen, Piotr Mydel, Jan Potempa

**Affiliations:** 1 Department of Microbiology, Faculty of Biochemistry, Biophysics and Biotechnology, Jagiellonian University, Krakow, Poland; 2 Broegelmann Research Laboratory, Department of Clinical Science, University of Bergen, Bergen, Norway; 3 Department of Rheumatology and Inflammation Research, Sahlgrenska Academy, University of Gothenburg, Gothenburg, Sweden; 4 Department of Human Developmental Biology, Jagiellonian University College of Medicine, Krakow, Poland; 5 Kennedy Institute of Rheumatology, University of Oxford, London, United Kingdom; 6 Institute of Dental Research, Westmead Centre for Oral Health and Westmead Millennium Institute, Sydney, Australia; 7 Department of Oral Biology, Faculty of Dentistry, University of Sydney, Sydney, Australia; 8 University of Louisville School of Dentistry, Center for Oral Health and Systemic Diseases, Louisville, Kentucky, United States of America; Yale University, United States of America

## Abstract

Rheumatoid arthritis and periodontitis are two prevalent chronic inflammatory diseases in humans and are associated with each other both clinically and epidemiologically. Recent findings suggest a causative link between periodontal infection and rheumatoid arthritis via bacteria-dependent induction of a pathogenic autoimmune response to citrullinated epitopes. Here we showed that infection with viable periodontal pathogen *Porphyromonas gingivalis* strain W83 exacerbated collagen-induced arthritis (CIA) in a mouse model, as manifested by earlier onset, accelerated progression and enhanced severity of the disease, including significantly increased bone and cartilage destruction. The ability of *P. gingivalis* to augment CIA was dependent on the expression of a unique *P. gingivalis* peptidylarginine deiminase (PPAD), which converts arginine residues in proteins to citrulline. Infection with wild type *P. gingivalis* was responsible for significantly increased levels of autoantibodies to collagen type II and citrullinated epitopes as a PPAD-null mutant did not elicit similar host response. High level of citrullinated proteins was also detected at the site of infection with wild-type *P. gingivalis*. Together, these results suggest bacterial PAD as the mechanistic link between *P. gingivalis* periodontal infection and rheumatoid arthritis.

## Author Summary

Clinical and epidemiological data indicates that chronic periodontal disease (PD), one of the most prevalent infectious inflammatory disease of mankind, is linked to systemic inflammatory diseases such as cardiovascular diseases (CVD), rheumatoid arthritis (RA) and chronic obstructive pulmonary disease (COPD). Nevertheless, the causative mechanisms of association between PD and chronic inflammatory diseases are very poorly understood. Recent findings suggest a causative link between periodontal infection and rheumatoid arthritis *via* bacteria-dependent induction of a pathogenic response to citrullinated epitopes. In present study we show that infection with viable periodontal pathogen *Porphyromonas gingivalis* but not another oral bacterium (*Prevotella intermedia*), exacerbated CIA, as manifested by earlier onset, accelerated progression and enhanced severity of the disease, including significantly increased bone and cartilage destruction. The ability of *P. gingivalis* to augment CIA was dependent on the expression of a unique enzyme peptidylarginine deiminase, which converts arginine residues in proteins to citrulline. This knowledge may create new perspectives in the treatment and prevention of RA in susceptible individuals.

## Introduction

Rheumatoid arthritis (RA) and periodontal disease (PD) are two common chronic inflammatory diseases affecting humans with considerable consequences for public health and for the quality of life of affected individuals [1]. In the case of PD, inflammation is initiated and perpetuated by a subset of bacteria, including *Porphyromonas gingivalis*, which colonize the gingival sulcus and proliferate in the subgingival plaque. The resulting chronic inflammatory response by the host cause eventual destruction of the supporting structures of the teeth, leading to loss of the dentition in severe PD. In contrast, rheumatoid arthritis is an autoimmune disease with subsequent chronic inflammation responsible for bone and cartilage destruction within the joints [2], [3]. Antibodies against citrullinated proteins are known to be a specific marker that can be detected years before the onset of the disease and their presence and serum levels correlate strongly with disease severity. Protein citrullination is carried out by endogenous peptidyl-arginine deiminases (PADs). These enzymes catalyze the conversion of peptidyl-arginine to peptidyl-citrulline, which is essential for many physiological processes [4]. However, PAD-catalyzed protein citrullination also occurs under pathological inflammatory conditions like necrosis and has been linked to the breakdown of immune tolerance to citrullinated proteins leading to induction of RA in susceptible individuals [5], [6].

Although RA and PD differ in terms of their etiological mechanisms, a link between both diseases has been established in numerous clinical and epidemiological studies [7]. Specifically, compared to the general population, individuals with PD have an increased prevalence of RA and, conversely, PD is at least 2-fold more prevalent in RA patients [8]. Moreover, the clinical outcome of PD in RA patients is more severe than in non-RA individuals and is independent of age, gender, ethnicity, or smoking habits. Finally, therapeutic treatment of one disease ameliorates the signs and symptoms of the other [9], [10].

The non-causal character of the PD-RA association may be due to shared genetic and environmental risk factors, such as MHC class II HLA-DRB1 epitope and smoking, respectively [8]. Furthermore, the two diseases have common effector destructive mechanisms, including a similar set of effector inflammatory cells, proinflammatory cytokines, and other mediators that drive bone erosion. Recent findings indicate that infection with *P. gingivalis* precedes RA and that the bacterium is a likely factor in the initiation and maintenance of the autoimmune inflammatory responses that occur in this disease [11], [12]. In this respect, presence of *P. gingivalis* PAD (PPAD), an enzyme expressed by *P. gingivalis* but absent in other prokaryotes [13], may have a profound impact on the development and progression of RA via citrullination of proteins to generates neo-epitopes as hypothesized in several recent reviews [14]–[16]. This novel hypothesis was tested in the present work, in which the pathogenic outcome of collagen-induced arthritis (CIA) was investigated in mice infected with *P. gingivalis* wild-type or PAD-null isogenic strains.

## Results

### Impact of *P. gingivalis* infection on collagen-induced arthritis development

**Figure 1:**
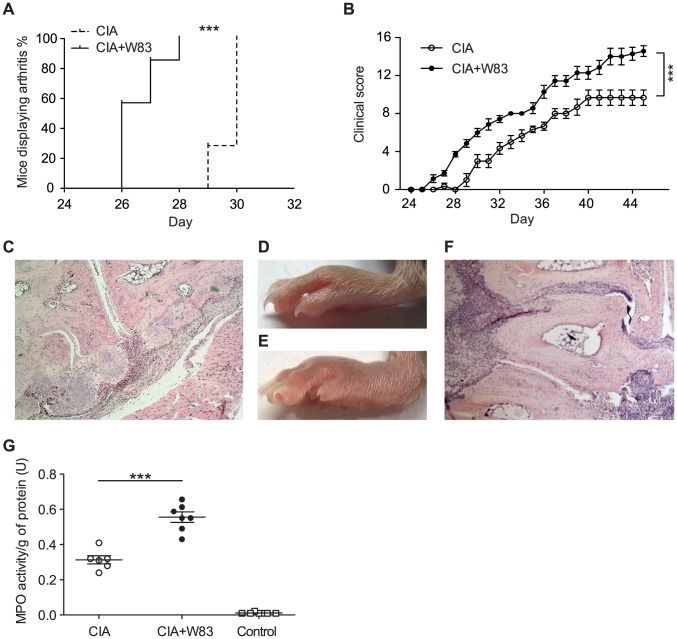
*P. gingivalis* exacerbation of CIA in DBA/1 mice. Mice (n = 7) were inoculated with 1×10^8^ *P. gingivalis* strain W83 cells and subsequently immunized with CII. (A) Kaplan-Meier plots showing differences in the clinical onset of arthritis (log-rank test, p<0.001) (n = 7). (B) Mean severity of arthritis in mice followed for 5–6 weeks after immunization. Two-way ANOVA with Bonferroni's post-test was used for the statistical evaluation. (n = 7). (C) H&E staining of the representative inflamed tarsal joints from CII- immunized DBA/1, and (F) mice infected with wild-type *P. gingivalis* strain W83 (D) Representative picture of the inflamed lower paw in CIA and (E) CIA after infection with wild type *P. gingivalis*. (G) Scatter dot plot showing MPO activity in 1 g of joint tissue extract prepared at day 45 after CII immunization. Horizontal bar and error bars represent the mean and SEM, respectively. Differences between groups were analyzed using Mann-Whitney U test (n = 20). *** denotes p<0.001.

To document that *P. gingivalis* can impact on the initiation, rate of progression, and severity of arthritis we have adopted the CIA model to quantify the contribution of infection with *P. gingivalis* in the disease process. Because of DBA/1 mice resistance to oral colonization by *P. gingivalis* we have employed the chamber model of infection [17]. To this end, sterile titanium wire coils were surgically implanted subcutaneously into mice. As part of the healing process, the coils were subsequently encased by fibrous tissues and the resultant hollow interior of the chambers became suitable for inoculation of live *P. gingivalis*. The open architecture of the wire chamber allows for a normal host immune response including immune cell infiltrates. The inoculum of 10^8^ CFU was selected on results of preliminary experiments to sustain an infection inside the chamber without systemic dissemination of bacteria. At specific time points, chamber contents were sampled via needle aspiration to assay for myeloperoxidase activity, level of protein citrullination and to confirm the presence of live bacteria. 29 days after subsequent collagen type II (CII) immunization, all animals inoculated with *P. gingivalis* wild-type strain W83 showed clinical signs of arthritis compared to only 28% of the control animals (p = 0.001, Fig. 1A). Mice infected with *P. gingivalis* had significantly increased severity of arthritis throughout the experiment (p<0.001 Fig. 1B, E, F) as compared to control (Fig. 1B, C, D). Histological evaluation at the end of the experimental period confirmed that *P. gingivalis* infection led to a 1.75-fold increase in synovitis (arthritis index 2.44±0.21, p<0.001). Moreover, bone and cartilage erosion was 1.76-fold higher (arthritis index 2.26±0.23, p<0.001) than in the CIA controls (synovitis 1.67±0.17 and erosions 1.28±0.23 respectively)(data not shown). By contrast, there were no significant differences in weight change and general health between control and infected animals.

Myeloperoxidase (MPO) activity, reflecting the number of infiltrating neutrophils, was determined in the joints of both groups of mice at day 45 of the experiment. Protein extracts from the excised joints showed significantly higher MPO levels in animals inoculated with *P. gingivalis* before CII immunization than in controls (p<0.001, Fig. 1G). The massive neutrophil influx at the sites of inflammation correlated with clinical observations of swelling and erythema.

### CIA augmentation is a feature of infection with live *P. gingivalis*

**Figure 2:**
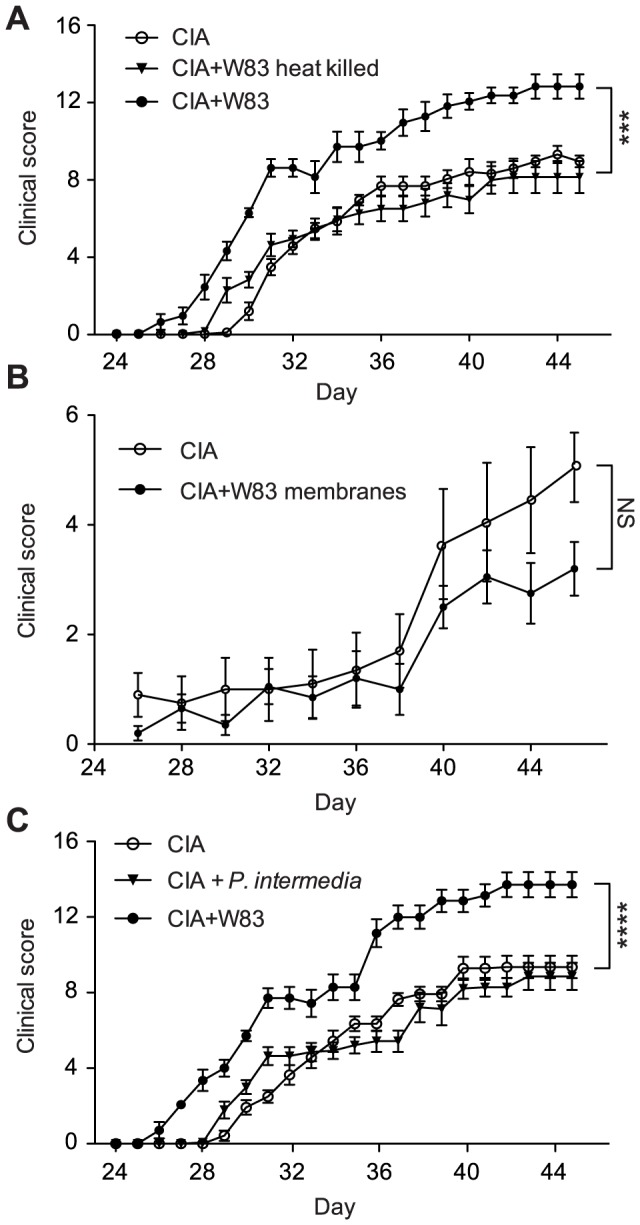
Aggravation of collagen-induced arthritis was specific to exposure to viable *P. gingivalis*. (A) Development of CIA at 5–6 weeks in DBA/1 mice (n = 7) were compared after exposure to viable *P. gingivalis* and heat-killed *P. gingivalis*; (B) Mice were immunized with *P. gingivalis* cell-envelope fractions (n = 10) 3 weeks prior to CII challenge and the development CIA were monitored at the 5–6 weeks period. (C) Mice were challenged with the periodontal pathogen *P. intermedia* strain 17 as compared to wild-type *P. gingivalis*. Points represent mean ± SEM. Two-way ANOVA with Bonferroni's post-test was used for the statistical evaluation (*** p<0.001, **** p<0.0001).

Interestingly, inoculation with heat-killed *P. gingivalis* did not affect either the rate or severity of CIA development suggesting a simple bacterial antigenic challenge is insufficient for the initiation of autoantibody production (Fig. 2A). Likewise, immunization with *P. gingivalis* cell-envelope fraction which is highly citrullinated [18] did not aggravate CIA development (Fig. 2B). Further, to investigate the possibility that infection with any periodontal pathogen will induce development of CIA, a second pathogen frequently associated with periodontal disease, *Prevotella intermedia*, was used as the infectious agent. Being considered as a secondary periodontopathogen [19] this gram-negative anaerobic bacterium is frequently found together with *P. gingivalis* in subgingival plaque [20], is associated with higher risk of coronary heart disease [21], has the ability to induce production of prostaglandin E2 which may contribute to bone resorption in RA and PD [22], and uses proteolytic enzymes (alike *P. gingivalis*) as main virulence factors [23], [24]. Moreover, *P. intermedia*-derived LPS stimulates strong proinflammatory reaction [25] and bacterium-derived DNA is frequently detected in synovial fluid of RA patients [26]. In the CIA mouse model, however, *P. intermedia* failed to cause aggravation of arthritis as compared to controls in which the mean clinical score at the end of experiment was 8.21 (combined score 57.5) and 9.28 (combined score 65), respectively (Fig. 2C). This is in contrast with *P. gingivalis* W83 inoculated mice which developed arthritis significantly earlier, with a higher prevalence and reaching a plateau clinical score at 12.86 (combined score 90) on day 40 (p<0.0001, Fig. 2C). Significantly there was no difference in bacterial survival in chambers and both live *P. gingivalis* and *P. intermedia* were detected in chamber fluid up to fifth day post-inoculation (data not shown). Taken together, these results clearly demonstrate that exacerbation of CIA is specific to infection with viable *P. gingivalis*.

### Impact of *P. gingivalis* PAD (PPAD) on CIA development

**Figure 3:**
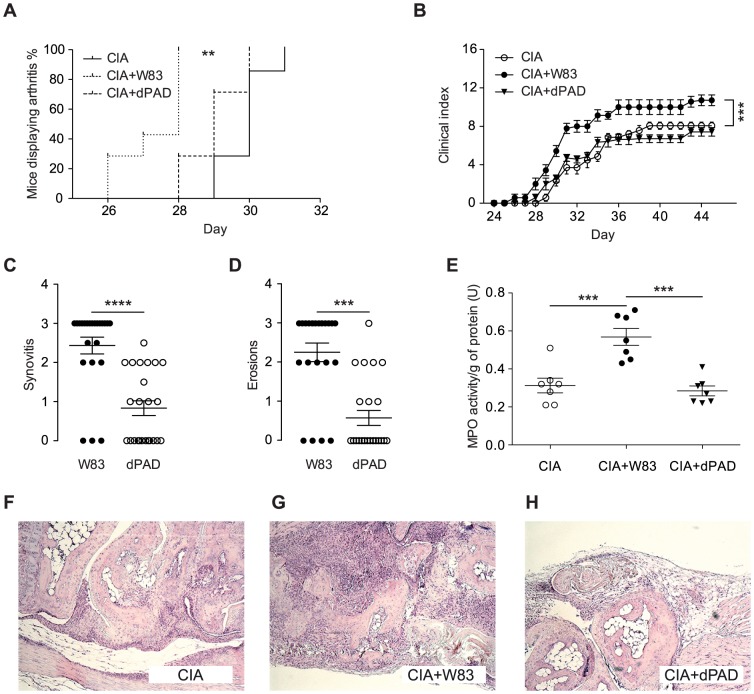
Aggravation of collagen-induced arthritis in DBA/1 mice was dependent on the production of *P. gingivalis* peptidylarginine deiminase (PPAD). ****** Mice (n = 7) were inoculated with 1×10^8^ CFU *P. gingivalis* wild-type strain W83 or PPAD-knockout strain (dPAD) and subsequently immunized with CII. (A) Kaplan-Meier plots displaying the clinical onset of arthritis (log-rank test, p = 0.002). (B) Mean severity of arthritis in mice followed for 5–6 weeks after immunization. (C) Histological signs of synovitis and (D) erosion on day 45 after CII immunization. (E) Scatter dot plot showing MPO activity in 1 g of joint tissue extract at day 45 after CII immunization. H&E staining of the representative inflamed tarsal joints from (F) CII- immunized DBA/1, (G) mice infected with wild-type *P. gingivalis* strain W83, and (H) isogenic dPAD mutant. Horizontal bar and error bars represent the mean and SEM, respectively. Statistical evaluation using two-way ANOVA with Bonferroni's post-test were employed for data in (B) and statistical differences between groups were analyzed using Mann-Whitney U test (C) or one way ANOVA (D). ** denotes p<0.01; ***, p<0.001; ****, p<0.0001.

**Figure 4:**
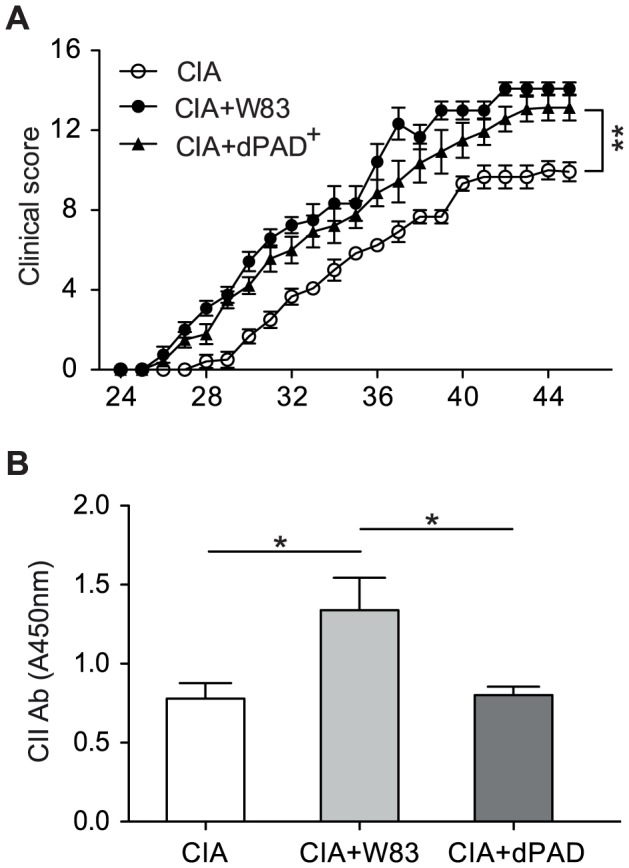
Aggravation of collagen-induced arthritis in DBA/1 mice was dependent on the production of *P. gingivalis* peptidylarginine deiminase (PPAD). (A) Development of arthritis in DBA/1 mice (n = 7) inoculated with *P. gingivalis* wild-type W83 strain or a PPAD-complemented mutant (dPAD^+^). Mean severity of arthritis in mice followed for 5–6 weeks after immunization. B) Serum levels of IgG antibodies against CII were determined by ELISA on day 45 after immunization. Horizontal bar and error bars represent the mean and SEM, respectively. Statistical evaluation using two-way ANOVA with Bonferroni's post-test (* p<0.05, ** p<0.01).

Considering the central role of citrullinated epitopes in the pathogenesis of RA, we investigated whether PPAD expression contributes to the enhancement of CIA by *P. gingivalis* infection. Towards this aim, an isogenic PPAD-knockout strain (dPAD) was used to inoculate the chambers prior to CII immunization. Again, no significant difference in survival of the dPAD mutant within the chamber in comparison to the parental *P. gingivalis* strain was observed. Nevertheless, on day 28 when, all mice inoculated with the wild-type W83 strain demonstrated clinical symptoms of arthritis, only 30% of the dPAD-infected animals were symptomatic (p = 0.002, Fig. 3A). In fact, the incidence of arthritis in dPAD mice was the same as in the CIA controls throughout the experiment (Fig. 3A, B). Histologically, signs of synovitis and erosions in dPAD mice were consistently similar to CIA controls (Fig.3 E, G), in contrast to heavy infiltrates observed in the W83 infected animals (Fig. 3F). Synovitis in the dPAD group (arthritis index: 0.83±0.19) was less than half of the W83 infected group (arthritis index: 2.43±0.21). The extent of bone and cartilage erosion in dPAD-infected animals (arthritis index: 0.58±0.19) was approximately one-quarter of those seen in the W83 group (arthritis index: 2.26±0.24) (Fig. 3C, 3D). Neutrophil infiltration quantity as estimated by MPO activity in joint tissues, was also significantly lower in the dPAD group (p<0.001, Fig. 3E). Complementation of the PPAD gene back into the dPAD strain (dPAD^+^) restored the ability of these bacteria to enhance arthritis development (p<0.01, Fig. 4A). This confirmed that PPAD is crucial for both the early development of CIA and the exacerbation of arthritis symptoms.

Finally, given the role of autoantibody production in the pathophysiology of CIA, we examined the ability of *P. gingivalis* infection to influence serum levels of specific anti-CII antibodies. In mice inoculated with W83, the serum levels of specific IgG against CII were significantly higher than in either dPAD or CIA control mice (p<0.05, Fig. 4B). Collectively, these data are consistent with the clinical and morphological findings and argue that the exacerbation of CIA by *P. gingivalis* infection is dependent on PPAD expression, which appears to stimulate autoantibody production.

### Effect of *P. gingivalis* on host protein citrullination levels

**Figure 5:**
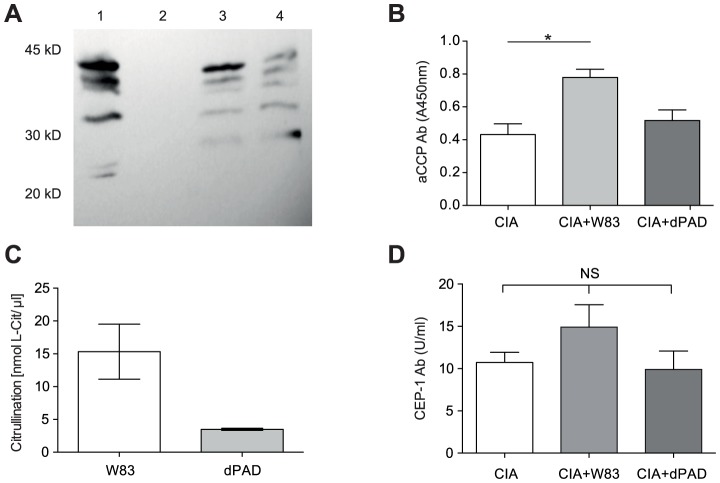
Inoculation with *P. gingivalis* strain W83 leads to citrullination *in vivo* and, in turn, production of antibody against citrullinated proteins (aCCP). (A) Subcutaneously implanted titanium chambers were inoculated with: 1) 1×10^8^ *P. gingivalis*, 2) PBS, 3) 5×10^7^ and 4) 1×10^7^ *P. gingivalis*. After 24 h, 20 µl of chamber fluid was collected and assayed for citrullinated proteins by immunoblotting with anti-modified citrulline antibody. B) Level of citrullinated proteins in chamber fluid 7 days post-inoculation. Pooled chamber fluid from 2 independent experiments was used. Horizontal bar and error bars represent the mean and SEM, respectively. C) Serum titers of aCCP IgG and D) IgG antibodies against CEP-1 were determined using an ELISA 45 days post-immunization with CII. Error bars represent the SEM. Statistical evaluation was carried out using one way ANOVA. * denotes p<0.05.

Since the autoimmune response to citrullinated proteins has been reported to have a significant impact on the development and progression of RA in experimental autoimmune models [17], both the chamber fluid and the serum of mice inoculated with *P. gingivalis* were assayed for the presence of citrullinated proteins and for antibodies specific to citrullinated epitopes, respectively. Using Western blot, citrullinated proteins were detected exclusively in the chamber fluid of animals inoculated with wild-type *P. gingivalis* and the intensity of citrullination was proportional to the inoculum dosage (Fig. 5A). In control animals injected with culture broth, neither the chamber fluid nor the sera contained citrullinated proteins. However, when the level of citrulline was determined by a colorimetric method, some citrullination was also detected in chambers inoculated dPAD but at lower levels than in mice infected with wild-type bacteria (Fig. 5B). This result shows that host PADs also contribute to citrullination but only at low levels in this model.

Since all attempts to directly detect citrullinated mouse fibrinogen in chamber fluid and serum by the Western blot using specific antibodies gave negative results (data not shown), we sought instead to detect the presence of antibodies reactive to citrullinated epitopes. To this end, we chose to measure the level of antibodies to citrullinated peptides (aCCP) and to specific enolase-derived citrullinated epitope (CEP-1) implicated in autoimmunity in RA [11]. In comparison to CIA control mice or CIA mice infected with dPAD mutant, there was a clear increase in the level of antibodies against CEP-1 in CIA mice infected with wild-type *P. gingivalis* (Fig. 5D). Although this increase was not statistically significant, the observed trend corroborates with a significantly higher serum level (1.9-fold) of antibodies to citrullinated peptides (aCCP) in mice inoculated with W83 as compared to either dPAD-infected or CIA control mice (p<0.05 Fig. 5C). This result suggests that *P. gingivalis*-induced protein citrullination within the chamber was related to the systemic humoral immune response to CIA within the joints.

Recently, we have found anti-PPAD antibody response is unique for RA patients suggesting that PPAD may constitute a neo-antigen in RA [27]. To assess if a similar immune response was elicited in mice infected with *P. gingivalis* we measured serum anti-PPAD antibody level using ELISA. In line with findings in RA patients, mice inoculated with wild-type *P. gingivalis* showed the presence of anti-PPAD antibodies but they were found at very low level (Fig. S1) making discussion about their importance for CIA aggravation speculative at this stage.

## Discussion

Across millennia, oral health status, as evaluated by tooth decay, has been a prognostic indicator of general health in humans. As early as 400 BC, Hippocrates noted the association with ‘rheumatism’ by describing a case of joint pain that was successfully treated by tooth extraction [12]. Within the last 20 years, this correlation has been strengthened clinically and epidemiologically but the mechanistic link between periodontitis and RA has remained elusive. Now, there is an increasing body of evidence that severity of periodontitis is not only related to progression of RA, but also that the profile of oral microbiota in patients with new onset RA is extremely similar to that in patients suffering from chronic RA [28], [29]. Here, for the first time, we demonstrate that infection with *P. gingivalis* not only exacerbates CIA but also appears to play a role in sensitizing animals to early disease development. The arthritis that occurred in mice infected with *P. gingivalis* was characterized by significantly greater bone and cartilage destruction in the affected joints. These clinical signs of arthritis manifested significantly earlier and were accompanied by a more severe disease course than in non-infected animals. Moreover, aggravation of these RA-associated pathologies was shown to be dependent on PPAD activity which either directly or indirectly via enhancement of inflammatory reaction and release of host PADs leads to generation of citrullinated neo-epitopes. Regardless of the mechanism the presented data implicates that PPAD, which among prokaryotes is unique to *P. gingivalis*, is a possible trigger of a pathogenic autoimmune response leading to RA development or at least may aggravate the course of the disease.

Both humoral and cellular immune responses have been shown to contribute to the pathogenesis of PD and RA. Activated neutrophils are primarily responsible for the characteristic tissue damage through the release of deleterious proteinases and pro-inflammatory cytokines [30]. Consistent with those observations, we found that neutrophil influx, as measured by MPO activity in tissue homogenates, was significantly higher in the affected joints of animals inoculated with *P. gingivalis*. It is important to mention that neutrophil presence was not a consequence of bacteremia, as the use of a variety of detection methods, including PCR-based techniques, failed to detect any bacteria in the joints of infected mice throughout the course of the experiment.

The early onset and aggravated progression of CIA occurred only in animals inoculated with viable *P. gingivalis*. Infection with *P. intermedia*, another important pathogen associated with periodontal diseases [31], exerted no effect on the course of CIA. Significantly, there were no observed differences in survival of *P. intermedia* and *P. gingivalis* in the subcutaneous chambers. Nevertheless, it cannot be excluded that *P. intermedia* fail to aggravate CIA because it elicited a weaker host inflammatory response as compared to *P. gingivalis*. Similar to infection with *P. intermedia*, inoculation with either heat-killed *P. gingivalis* or purified cell-envelope fraction from the same organism had no effect on either the rate or progression of CIA. Therefore, we hypothesized that the requirement for live *P. gingivalis* must be associated with active bacterial component(s) exerting a direct effect on the host that ultimately triggered an autoimmune reaction. This hypothesis also drew on the recent findings in which immunity to *P. gingivalis*, but not to *P. intermedia* (or *Fusobacterium nucleatum*), was shown to be significantly associated with the presence of RA-related autoantibodies in individuals at risk for the disease [32].

An increasing number of studies have pointed to post-translational modifications as one of the main factors in the ability of pathogens to breach immune tolerance [33]–[35]. Among these modifications, citrullination of endogenous proteins seems to be a key process in the initiation of autoimmune reactions [3], [36]. Citrullination normally plays a pivotal role in the formation of myelin and is crucial for normal structure of hair and skin. In humans, five PADs (PAD1–PAD4 and PAD6) are variably expressed in different tissues and these enzymes mediate deimination of Arg residues within proteins in a calcium-dependent manner [37]. However, aberrant citrullination has been observed in autoimmune diseases, among which, RA is a prime example [38]. It is unclear, however, whether citrullination in RA creates novel epitopes or uncovers cryptic ones in susceptible individuals. The potential autoantigens in RA that are efficiently citrullinated by human PADs include enolase, fibrinogen, vimentin, and CII [39]–[41].

Through its unique expression of PAD, *P. gingivalis* is the only prokaryote that is able to citrullinate proteins [42]. Not only is PPAD able to citrullinate the cell-envelope proteins of *P. gingivalis*, it also had been shown to modify host proteins *in vitro* [18]. The enzyme seems to also be immunogenic in mice as we were able to detect some antibodies against PPAD in animals inoculated with wild type strain W83. Nevertheless, it is unlikely that this response contributes to aggravation of CIA by *P. gingivalis* because the level of anti-PPAD antibodies was very low. Unlike human PAD, PPAD can efficiently modify C-terminal arginine and its activity is calcium-independent, which enables protein modification even in environments with low calcium concentrations. In our experiments, the inoculation of animals with either a highly citrullinated cell-envelope fraction or heat-killed bacteria carrying citrullinated proteins on their surface did not alter CIA development and progression. Therefore, we assume that PPAD generates non-self epitopes, specifically C-terminal citrulline residues on host proteins cleaved at Arg-Xaa peptide bonds by gingipains [18] and thereby contributes to CIA pathology. To test this hypothesis, an isogenic *P. gingivalis*-PAD knockout strain (dPAD) was created. Inoculation of animals with dPAD prior to immunization with CII had no influence on clinical CIA development and progression. Importantly, there was no significant difference in survival of the dPAD mutant and the parental wild-type strain within implanted chambers, in correlation with the same rate of growth *in vitro* of both strains (Fig. S2). Histologically, dPAD inoculation had no effect on the degree of synovitis, erosions, or neutrophil influx into the joints as compared to CIA controls. This is in complete contrast to animals infected with the live W83 strain, highlighting the fact that PPAD probably contributes significantly towards the pathogenesis of the disease.

Antibodies against citrullinated proteins are known to be a specific marker for RA [43], [44] and can be detected years before the onset of the disease. In addition, their presence and serum levels correlate strongly with disease severity [45]. Importantly, in humans, PD in non-smoking untreated RA patients is associated with high titers of antibodies against citrullinated proteins (aCCP) [46]. Nevertheless, the role and/or the presence of aCCP antibodies in CIA is highly controversial. Meaningfully, however, our results showed a significant increase in aCCP antibody titers only in the serum of animals infected with the parental *P. gingivalis* strain W83, whereas animals inoculated with isogenic dPAD, heat-killed bacteria, or cell-envelope fraction, aCCP antibodies remained at similar levels to the CIA controls. Significantly, the increase in aCCP antibodies was associated with high concentrations of citrullinated proteins in the chamber fluid of mice infected with strain W83 and correlated with increased level of antibodies specific for human enolase-derived citrillinated epitope (CEP-1) which cross-reacts with the equivalent epitope of *P. gingivalis*-derived enolase. This bacterial epitope was implicated in induction of autoimmune reaction in RA [41].

It should be kept in mind the essential differences in citrullinated antigenic epitopes generated by endogenous PADs and PPAD. In particular, *P. gingivalis* citrullination of surface proteins is dependent on the action of Arg-gingipains proteases expressed in abundance by the organism since Arg-gingipain-null mutants are devoid of citrullination. This concerted action facilitated by co-localization of both enzymes in the outer membrane also targets host proteins to generate polypeptides with citrullinated Arg located at the C-termini. Antibodies against such epitopes may not recognized internal citrullines generated by PADs and vice versa, but they may still bind to CCP system with sequences optimized to detect a wide range of host antibodies against different citrullinated epitopes. Although we were not able to specifically identify the modified proteins, high levels of citrullinated proteins in the chamber fluid suggests that PPAD contributes, at least partially, to the characteristic intensive citrullination of proteins in the periodontal stroma in PD [47]. Together, it is tempting to hypothesize host proteins citrullinated by PPAD-producing *P. gingivalis* may contribute to a breach of immune tolerance in susceptible individuals that subsequently triggers an inappropriate immune response that exacerbates CIA. This knowledge may create new perspectives in the treatment and prevention of RA.

## Materials and Methods

### Mice

Animal experiments were performed on 6- to 8-weeks old male DBA/1 mice that were obtained from Medical College of Jagiellonian University and Taconic (Europe A/S, Ry, Denmark), respectively.

### Bacterial strains

*Porphyromonas gingivalis* strain W83 and *Prevotela intermedia* strain 17 were grown on blood agar plates in an anaerobic chamber with 85% N_2_, 5% H_2_ and 10% CO_2_. Isogenic PPAD-knockout strain (dPAD) of P. gingivalis W83 and complement mutant (dPAD^+^) [18] were grown under the same conditions with the addition of erythromycin at a concentration of 5 µg/ml. After incubation at 37°C for 5 days, bacterial cells were inoculated into enriched Schoedler broth (Becton Dickinson), supplemented with hemin (5 mg/ml) and menadione (0.5 mg/ml) and grown overnight in anaerobic conditions.

Prior to inoculation, bacteria were washed three times with phosphate-buffered saline (PBS) and resuspended in fresh culture medium. Bacterial cell counts were determined using a spectrophotometer, where the optical density of 1.0 at 600 nm corresponded to 1×10^9^ CFU per/ml.

### Experimental protocols of *in vivo* studies

Two separate sets of experiments regarding impact of *P. gingivalis* and citrullination were conducted. To elucidate importance of live bacteria, subcutaneous chamber model was employed in our experiments as an established *P. gingivalis* infection model that creates a niche inside hosts for bacterial colonization [17]. To this end titanium wire coils (10 mm×5 mm) were implanted subcutaneously in the dorso-lumbar region of each mouse following anesthesia. Incisions were closed using 4.0 G silk sutures and the animals were allowed to rest for 14 days to heal completely to allow for the formation of a fibrous capsule surrounding the coils, thereby, creating chambers into which bacteria or cell-envelope of *P. gingivalis* could be inoculated. We employed this model instead oral gavage model (oral infection) because mice are not natural host for *P. gingivalis*. Further, the CIA model requires use of DBA/1 animals, which are resistant to *P. gingivalis* colonization after oral challenge. We have performed a few trial experiments using live *P. gingivalis* (W83 strain) in the oral infection model but we were not able to detect viable *P. gingivalis* after 24 h post-challenge. CII immunization was performed 14 days after bacteria inoculation. To study the influence of auto-citrullinated proteins of *P. gingivalis*, mice were immunized with 50 µg of the cell envelope fraction 2 weeks prior to CIA.

### Chamber inoculation with *P. gingivalis*

DBA/1 mice were divided into experimental groups of at least 7 mice and each animal was implanted with a coiled wire chamber at the dorsolateral aspect. Animals were allowed to recover from the surgical procedure for 14 days prior to being challenged with bacteria. Bacteria were inoculated with 10^8^ CFU (0.1 ml at 10^9^ CFU/ml) into the lumen of the sealed chamber with a 25-G needle through the disinfected skin. Sterilized 0.1 mL of the same culture medium was used to sham inject the control chambers. The course of infection was monitored clinically. Two weeks after chamber injection with *P. gingivalis*, mice were immunized with CII

### CIA

Immunization and arthritis evaluation was performed as described [48] with modification. DBA/1 mice were injected *i.d.* at the base of the tail with 100 µg CII (Chondrex) in CFA. Booster immunization containing 100 µg of CII in IFA was administered 21 days after the priming.

### Clinical evaluation of arthritis

All DBA/1 mice were inspected at regular intervals to ascertain the presence of arthritis. To evaluate the intensity of arthritis, a clinical scoring system of 0–4 points for each paw was used: 0, no sign of inflammation; 1, mild swelling and/or erythema; 2, moderate swelling and erythema; 3, marked swelling and erythema; 4, extreme erythema and swelling. Clinical index was constructed by adding the scores from all four limbs for each animal.

### Histological examination

All four paws from DBA/1 mice in the first experiment were excised at the termination of the experiment (45 days after CII immunization). Tissue sections were stained with hematoxylin/eosin and synovitis and erosion of bone/cartilage were evaluated by a blinded examiner. A histological scoring system was used as follows: 1, mild; 2, moderate; and 3, severe synovitis and joint damage. Knee joints, ankles, elbows, and wrists were inspected, and a mean score from all of the inspected paws in each animal was calculated.

### Purification of *P. gingivalis* cell envelope

Bacterial cells were collected from culture medium by centrifugation, resuspended in 50 mM N-(2-hydroxyethyl) piperazine-N′-(2-ethanesulphonic acid), pH 7.0, and sonicated in an ice bath at 1500 Hz for five 1 minute cycles (Sonics and Materials). Unbroken cells and large debris were removed by centrifugation and the cloudy supernatant was clarified by ultracentrifugation (32,500× g, 60 min, 4°C). The resulting clear supernatant was regarded as the membrane-free cell extract. The pellet containing the outer membrane, peptidoglycan and the inner membrane was resuspended in the same buffer and referred to as cell envelope fraction.

### Detection of citrullinated proteins in the chamber fluid

Up to 50 µl of chamber fluid was aspirated on day 1 after inoculation and centrifuged to remove bacteria. Citrullinated proteins were detected using an anti-citrulline (modified) detection kit (Upstate/Millipore) in accordance with the manufacturer's instructions.

### Measurements of aPPAD antibodies in mouse sera

CEP-1 was diluted at 10 µg/ml in coating buffer (50 mM carbonate buffer, pH 9.5), and PPAD was diluted at 5 µg/ml in coating buffer. Costar 96-well ELISA plates were coated with 50 µl/well antigen solution and incubated overnight at 4°C. Wells were washed 4× with PBS-Tween (0.05%) and blocked with 2% BSA in PBS for 5 hours at room temperature. Mouse serum was diluted 1∶50 in RIA buffer (1% (w/v) BSA, 350 mM NaCl, 10 mM Tris-HCl (pH 7.6), 1% (v/v) Triton X-100, 0.5% (w/v) Na-deoxycholate, 0.1% (w/v) SDS), added in duplicates, and incubated overnight at 4°C. Plates were washed as described above and incubated with peroxidase-conjugated rabbit anti-mouse IgG (Dako) (1∶5000) in RIA-buffer for 1.5 hours at room temperature. After a final wash, bound antibodies were detected with tetramethylbenzidine substrate (TMB) (KPL, Gaithersburg, MD). The reaction was stopped after 5 minutes by the addition of 1M H_2_SO_4_ and absorbance measured at 450 nm. All OD values are given following the subtraction of background for each plate (coating buffer alone). When the subtracted values were less than zero, a value of zero was given to the respective sample.

### Determination of *P. gingivalis* presence in inflamed joints

To verify the presence or absence of *P. gingivalis* in the inflamed joint tissue we have employed a standard cultivation method and PCR to specifically amplify 16S RNA of *P. gingivalis*. The forward (sense) primer 5′-CGTGCCAGCAGCCGCGGTAATACG-3′ and the reverse (antisense) primer 5′-TACATAGAAGCCCCGAAGGAAGAC-3′) were used. PCR amplification was carried out using the following conditions: 40 cycles of denaturation at 95°C for 30 sec, annealing at 60°C for 30 sec, and elongation at 72°C for 1 min. The PCR products were separated by agarose gel electrophoresis and visualized.

### Determination of joint myeloperoxidase (MPO) content

Joint tissues were homogenized in 0.5% hexadecyltrimethylammonium bromide pH 6.0 (50 mg of tissue/ml). The homogenates were freeze – thawed 3 times, centrifuged at 40,000× g and 0.1 ml aliquots were mixed with 2.9 ml phosphate buffer (pH = 6.0) containing H_2_O_2_ and 0.167 mg/ml o-dianisidine dihydrochloride (Sigma Aldrich). Absorbance was measured at 460 nm. MPO activity was expressed in units per protein concentration (U/g of protein).

### Determination of citrulline content and PPAD activity in chamber fluid

The presence of citrulline was determined using the modified Boyde and Rahmatullah method [49], based on the chemical modification of citrulline side-chains and colorimetric detection of their derivatives. Briefly, 1 µl of chamber fluid was diluted with 9 µl of PBS and mixed with 0.1 M Tris, 5 mM DTT in a 96-well followed by 10 µl of 5 M HClO4. The reaction was developed by the addition of 150 µl freshly prepared 1∶2 mixture of solution A (0.5% diacetyl monoxime, 0.01% thiosemicarbazide in water) and B (0.25 mg/ml FeCl3, 24.5% H2SO4, 17% H3PO4 in water). The plates were incubated at 110°C for 17 minutes. Color development was measured using a SpectraMax microplate absorbance reader (Molecular Devices, CA, USA) at 535 nm. Quantity of citrulline in the samples was calculated via a standard curve using free L-citrulline.

### Measurement of serum anti-CII, anti CEP-1 and anti-citrullinated peptide (aCCP) antibody levels

Serum samples were collected on day 45 post-immunization and level of CII-specific IgG was measured using a mouse anti-type II collagen IgG assay kit. Levels of aCCP in sera were measured using Immunoscan RA Anti-CCP Test Kit with modifications. Peroxidase-conjugated rabbit anti-mouse IgG specific for gamma chains antibodies were used for detection (Dako A7S, Denmark).

### Statistical analysis

Statistical comparison analyses were performed using GraphPad Prism (GraphPad, San Diego, CA). All results presented in the text are means ± standard error of the means. Differences between 2 groups were tested for statistical significance using Mann-Whitney U test and the log-rank test was used for comparing Kaplan-Meier curves. 2-way ANOVA and one way ANOVA with Bonferroni posttest were used for comparison of multiple groups. A *p* value of <.05 was considered statistically significant

### Ethical statement

All studies were performed in accordance with European Union regulations for the handling and use of laboratory animals. The protocols were approved by the institutional Animal Care and Use Committees (Jagiellonian University, Krakow, Poland, permit No. 94/2009) and Animal Research Committee of University of Gothenburg.

## Supporting Information

Figure S1 Serum levels of IgG antibodies against PPAD were quantified with ELISA on day 45 post-immunization. Horizontal bar and error bars represent the mean and SEM, respectively.
(TIF)

Figure S2 Comparison of the growth curves of *P. gingivalis* wild type W83 and its isogenic dPAD mutant. Cells were grown in TSB medium. Representative growth curves are shown. One-milliliter aliquots were taken, and cell density at OD 600 nm was measured over a 36 h period.
(TIF)
