# Supplementary figures and images for: Porphyromonas gingivalis Facilitates the Development and Progression of Destructive Arthritis through Its Unique Bacterial Peptidylarginine Deiminase (PAD)
Source: PLoS Pathog. 2013 Sep 12;9(9):e1003627. doi: 10.1371/journal.ppat.1003627 (PMC3771902; doi:10.1371/journal.ppat.1003627)

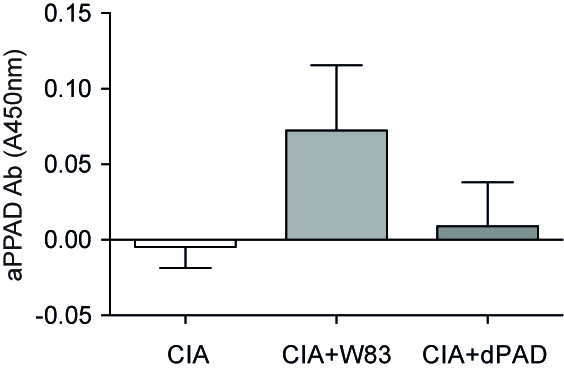

Supplement: Figure S1 — Serum levels of IgG antibodies against PPAD were quantified with ELISA on day 45 post-immunization. Horizontal bar and error bars represent the mean and SEM, respectively. (TIF) [file ppat.1003627.s001.tif]

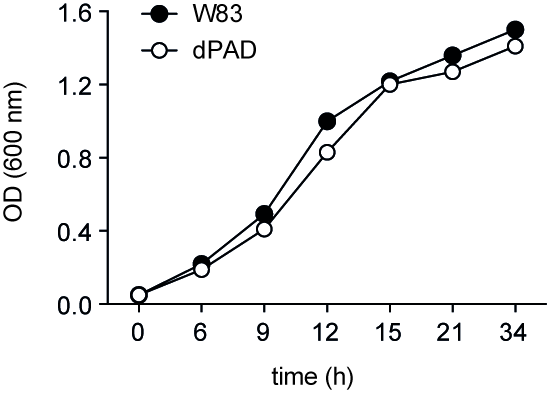

Supplement: Figure S2 — Comparison of the growth curves of P. gingivalis wild type W83 and its isogenic dPAD mutant. Cells were grown in TSB medium. Representative growth curves are shown. One-milliliter aliquots were taken, and cell density at OD 600 nm was measured over a 36 h period. (TIF) [file ppat.1003627.s002.tif]
